# 8^th^ international conference on management and rehabilitation of chronic respiratory failure: the long summaries – part 1

**DOI:** 10.1186/s40248-015-0026-z

**Published:** 2015-10-06

**Authors:** Nicolino Ambrosino, Richard Casaburi, Alfredo Chetta, Enrico Clini, Claudio F. Donner, Michael Dreher, Roger Goldstein, Amal Jubran, Linda Nici, Caroline A. Owen, Carolyn Rochester, Martin J. Tobin, Guido Vagheggini, Michele Vitacca, Richard ZuWallack

**Affiliations:** Weaning and Pulmonary Rehabilitation Unit, Auxilium Vitae Rehabilitation Centre, Volterra, PI Italy; Los Angeles Biomedical Research Institute at Harbor-UCLA Medical Center, Torrance, CA 90502 USA; Respiratory Diseases & Lung Function Unit, Department of Clinical & Experimental Medicine, University of Parma, Parma, Italy; DU of Medical and Surgical Sciences, University of Modena, Ospedale Vill Pineta, Pavullo n/F, Modena Italy; Mondo Medico, Multidisciplinary and Rehabilitation Outpatient Clinic, Borgomanero, NO Italy; Department of Cardiology, Pneumology, Angiology and Intensive Care Medicine, University Hospital Aachen, Aachen, Germany; West Park Healthcare Centre, University of Toronto, Toronto, Canada; Division of Pulmonary and Critical Care Medicine, Edward Hines Jr. Veterans Affairs Hospital and Loyola University of Chicago Stritch School of Medicine, Hines, IL 60141 USA; Pulmonary and Critical Care Section, Brown University, Providence Veterans Affairs Medical Center, Providence, RI USA; Division of Pulmonary and Critical Care Medicine, Brigham and Women’s Hospital/Harvard Medical School, Harvard Institutes of Medicine Building, 77 Avenue Louis Pasteur, Room 855B, Boston, MA 02115 USA; Yale University School of Medicine, VA Connecticut Healthcare System, New Haven, CT USA; Respiratory Unit and Weaning Center, Salvatore Maugeri Foundation, IRCCS Institute of Lumezzane, Lumezzane, BS Italy; Pulmonary and Critical Care, St Francis Hospital, Hartford, CT 06106 USA; Lovelace Respiratory Research Institute, Albuquerque, NM USA

**Keywords:** Asthma and COPD therapy, COPD exacerbations

## Abstract

This paper summarizes the Part 1 of the proceedings of the 8^th^ International Conference on Management and Rehabilitation of Chronic Respiratory Failure, held in Pescara, Italy, on 7 and 8 May, 2015. It summarizes the contributions from numerous experts in the field of chronic respiratory disease and chronic respiratory failure. The outline follows the temporal sequence of presentations.

This paper (Part 1) includes sections regarding: Advances in Asthma and COPD Therapy (Novel Therapeutic Targets for Asthma: Proteinases, Blood Biomarker Changes in COPD Patients); The problem of Hospital Re-Admission following Discharge after the COPD Exacerbation (Characteristics of the Hospitalized COPD Patient, Reducing Hospital Readmissions Following COPD Exacerbation).

## Background

This paper summarizes the Part 1 of the proceedings of the 8^th^ International Conference on Management and Rehabilitation of Chronic Respiratory Failure, held in Pescara, Italy on 7 and 8 May, 2015. It summarizes the contributions from numerous experts in the field of chronic respiratory disease and chronic respiratory failure. The outline follows the temporal sequence of presentations.

### Recent developments in Asthma and COPD Therapy

#### Rationale

The optimal therapy of patients with obstructive airways disease requires a thorough understanding of the mechanisms underlying the diseases and blending pharmacologic and nonpharmacologic therapies to address the primary disease processes, systemic effects, and co-morbidities.

### Novel therapeutic targets for asthma: Proteinases (Caroline A. Owen)

#### Key points

1. Proteinases produced by both host and non-host cells contribute to the pathogenesis of asthma.
2. Among the host cell-derived proteinases linked to asthma, metalloproteinases and chitinases (or chitinase-like proteins) have been strongly linked to asthma pathogenesis.
3. Non-host sources of proteinases that contribute to the disease include fungi, insects, and bacteria.
4. Proteinases can either promote or limit the progression of allergen-induced airway inflammation and remodeling by regulating the activity of mediators of inflammation, leukocyte apoptosis, mucus metaplasia, TH2 airway inflammation, or airway remodeling processes.
5. Host-derived metalloproteinases have potential to be therapeutically targeted to improve the morbidity and mortality associated with asthma.

#### Background

Asthma is the most common chronic respiratory disease, and is the 5^th^ most common cause of death due to respiratory causes [1, 2]. Asthma severity reflects genetic and environment influences. When a genetically-susceptible individual inhales allergens, this may induce a TH2-type immune response in the airways with release of IgE from B-lymphocytes which triggers mast cell degranulation. Release of mast cell mediators induces the recruitment of eosinophils, TH2 lymphocytes, dendritic cells, and monocytes to the airways [3]. This inflammatory response is associated with structural changes in the airway including mucus metaplasia, sub-epithelial fibrosis, smooth muscle cell hypertrophy and hyperplasia, and angiogenesis. Proteinases derived from both host cells and non-host cells (e.g., fungi, insects, and bacteria) have been linked to asthma by: 1) studies showing changes in the expression of proteinases in blood or lung samples from asthma cases versus controls; and 2) studies of mice either over-expressing or deficient in proteinases in models of allergen-induced airway inflammation. Proteinases promote or limit the development of all pathologies observed in asthmatic airways. The section below will discuss the proteinases that have been implicated in asthma pathogenesis, their activities in regulating disease, and the potential for targeting proteinases as a novel therapeutic strategy in asthma.

#### Host cell proteinases and asthma

Among host cell proteinases linked to asthma, members of the metalloproteinase (MP) superfamily [including matrix metalloproteinases (MMPs) and proteinases with a disintegin and a metalloproteinase domain (ADAMs] have been most strongly implicated in asthma pathogenesis.

##### MMPs

MMPs are a family of 24 proteinases (in humans) having multiple domains including a catalytic domain with an active site zinc atom. [4] MMPs degrade extracellular matrix (ECM) proteins in the lung, and also play crucial roles in regulating airway inflammation and remodeling by cleaving cytokines and activating latent growth factors. Most is known about the expression and activities of MMPs −2, −7, −9, and −12 in asthma.

##### MMP-2 and −9 (gelatinase A and gelatinase B)

MMP-2 is produced by airway epithelial cells, macrophages, T-cells, and fibroblasts. MMP-9 is produced by the same cells and also by eosinophils, polymorphonuclear cells, monocytes, and dendritic cells. MMP-9 and −2 are elevated in blood and lung samples from asthma patients [5]. MMP-9 sputum levels are higher in patients with acute versus stable asthma, [6] and increase significantly after challenge with house dust mite protein in patients with allergic asthma [7]. Plasma MMP-9 levels are also elevated during acute asthma attacks [8, 9]. Mice deficient in MMP-2 and/or MMP-9 that have been sensitized and challenged with allergens die from asphyxia. These mice also have increased leukocyte counts in the airway walls but reduced leukocyte numbers in the airway lumens [10] when compared with allergen-treated wild type (WT) mice. MMP-2 and −9 promote resolution of airway inflammation by cleaving and activating cytokines in the airways thereby creating chemokine gradients that promote egress of leukocytes from the airway walls into the lumen where they are removed by phagocytes such as macrophages [11]. In chronic allergen challenged models, MMP-9 ^−/−^ mice have reduced sub-epithelial fibrosis indicating that MMP-9 promotes sub-epithelial fibrosis [12] which may be due to MMP-9 activating latent growth factors such as transforming growth factor-β [13].

##### MMP-7 (matrilysin)

MMP-7 is expressed by airway activated epithelial cells and macrophages. MMP-7 expression is increased mainly in airway epithelial cells in asthmatics, and MMP-7 levels are increased in nasal washing after ragweed allergen challenge [14]. Airway epithelial MMP-7 expression is increased in mice challenged with cockroach allergen. CRA-challenged MMP-7 ^−/−^ mice have reduced airway hyper-responsiveness (AHR) to aerosolized acetylcholine challenges and reduced allergic airway inflammation (AAI) compared with CRA-challenged WT mice [14] indicating that MMP-7 limits AHR and AAI in mice [14]. MMP-7 increases AHR and AAI in mice by increasing the expression of interleukin-25 (IL-25) and also by proteolytically activating IL-25 which induces the differentiation of TH2 lymphocytes and increases their production of TH2 cytokines. MMP-7 also reduces the number of T-regulatory lymphocytes in the airways of mice which restrain the activation of adaptive immune responses [14].

##### MMP-12 (metallo-elastase)

MMP-12 is produced by activated macrophages and are increased in asthmatics [15]. Allergen-treated MMP-12 ^−/−^ mice have reduced AAI and AHR associated with reduced lung levels of chemokine (C-C) ligand-3 (CCL3), CCL2, tumor necrosis factor-α and IL-5. These results indicate that MMP-12 increases AAI in mice likely by increasing lung levels of pro-inflammatory cytokines [16].

##### ADAMs

ADAMs are a family of >30 multi-domain proteinases so called because they have a disintegrin and a metalloproteinase domain [17]. They are type-I transmembrane proteinases expressed on cell surfaces. Most ADAMs have a MP domain, a disintegrin domain (which binds to integrins to regulate cell adhesion and migration), an epithelial growth factor (EFG)-like-cysteine rich domain, and a cytoplasmic tail which can regulate cell signaling [17]. ADAM33 and ADAM8 have been strongly linked to asthma.

##### ADAM33

ADAM33 was the first gene to be linked to asthma. In 2002, single nucleotide polymorphisms (SNPs) in the ADAM33 gene were found to be significantly associated with asthma and bronchial hyper-responsiveness [18]. ADAM33 is expressed by smooth muscle cells and fibroblasts but not by epithelial cells or leukocytes. Studies of Adam33 ^−/−^ mice treated with allergens found that Adam33 does not regulate AAI or AHR in mice [19]. Based upon its expression profile, ADAM33 may regulate airway remodeling in asthmatics [20] especially as it stimulates endothelial cell proliferation [21] and thus may increase angiogenesis in asthmatic airways.

##### ADAM8

ADAM8 is expressed by all leukocytes except for T-cells and airway epithelial cells. While asthmatics and controls have similar ADAM8 expression in airway epithelium, ADAM8 expression is reduced in airway leukocytes from asthmatic subjects versus controls [22]. Unlike ADAM33, SNPs in the ADAM8 locus have not been liked to asthma risk or phenotypes [23]. Two studies of Adam8 ^−/−^ mice and mice over-expressing ADAM8 treated with allergens reported that ADAM8 has anti-inflammatory activities [22, 24]. ADAM8 was reported to increase activation of the intrinsic apoptosis pathways in eosinophils and macrophages and reduce the half life of these cells in the airways [22]. However, two studies reported that ADAM8 promotes AAI by either increasing the migration of T-cells or eosinophils into the airways [25] or increasing dendritic cell numbers in the airways and airway levels of CCL11 (a chemokine for eosinophils) and CCL22 (a chemokine for monocytes, dendritic cells and activated T-cells) [26]. Whether ADAM8 contributes to airway remodeling processes in asthma is not clear. However, ADAM8 has an active MP domain and has potential to regulate sub-epithelial fibrosis or mucus metaplasia by proteolytically regulating the biologic activities of growth factors and ligands for the epithelial growth factor receptor [27].

##### Chitinases and chitinase-like proteins

Chitinases are proteinases that degrade chitin, an essential component in the exoskeleton of insects and parasites. Chitin is made of repeating units of β-(1–4)-poly-*N*-acetyl-d-glucosamine and is the second-most-abundant polysaccharide in nature. Humans and rodents express chitinases (as part of anti-parasite response) and also chitinase-like proteins that lack enzyme activity such as chitinase-like protein-1 (CHI3L1 or YKL-40) [28]. Lung and blood levels of these proteins are increased in asthmatics and allergen-treated mice. For example, YKL-40 expression in airway epithelium is strongly increased in asthmatics and its levels are correlated directly with asthma severity [29, 30]. Serum YKL-40 levels correlate directly with sup-epithelial thickness in asthmatic airways [29]. Polymorphisms in the acidic mammalian chitinase (AMC) gene are associated with asthma in humans [31]. YKL-40 promotes AAI in mice by inhibiting T-cell, macrophage, and eosinophil apoptosis/cell death, stimulating dendritic cell accumulation and activation, and inducing alternative macrophage activation [32]. AMC expression is increased in the airways of allergen-treated mice [28]. Delivering a blocking antibody to AMC to allergen-treated mice reduces IL-13 induced AAI and AHR indicating that AMC drives TH2-type inflammation in allergen-exposed airways [32].

##### Non-host proteinases

Proteinases produced by fungi, insects, and bacteria can promote TH2 inflammation. Common household fungi are a major source of active proteinases present in household dust and the main proteinase activity in dust resides in an ~85 kD multimer of aspergillo-pepsin I secreted by the *Aspergillus* genus [33]. Aspergillus-derived proteinases can serve as adjuvants in asthmatic airways [34]. Aspergillus fugal spores are inhaled, germinate in the airways, and release proteinases that are required for the expression of robust allergic disease which in turn enhances the clearance of fungi from the airways via the production of IL-13 and IL-5 and the development of eosinophilic inflammation. Aspergillus proteinases may promote TH2 airway inflammation by cleaving receptors on epithelial cells such as CD23 (a low affinity IgE receptor) and protease activated receptor-2 (PAR-2); a receptor which when cleaved by proteinases auto-activates itself to increase signaling in airway epithelial cells [35]. Aspergillus-derived proteinase may also cleave fibrinogen in extracellular fluids, and the cleavage products generated can activate toll-like receptor-4 (TLR4) on airway epithelial cells. Epithelial cells, thus activated, release products that promote TH2 airway inflammation such as MMP-7, IL-25, thymic stromal lymphopoetin, and complement component C3 [34]. In addition, serine proteinases produced by cockroaches, fungi, and amoeba also activate PAR2 on epithelial cells to promote AAI in experimental animals [36–38].

##### Proteinases as therapeutic targets in asthma

Among the proteinases linked to asthma, metalloproteinases (MPs) have been most strongly linked to asthma pathogenesis and could be targeted therapeutically. However, some MPs have beneficial activities in mice with asthma (MMP-2, MMP-9, and possibly ADAM8). Thus, it will be crucial to limit the activities of only MPs that promote asthma development (MMP-7, MMP-12, and possibly ADAM33). Small molecule metalloproteinases inhibitors (MPIs) have developed for other diseases, [39, 40] but are unlikely to be useful for asthma patients because current MPIs are not very selective as the active sites of MPs are similar. Moreover, MPIs tested for other diseases were associated with severe side effects (musculoskeletal syndrome) due to off target inhibition of ADAMs and ADAMs with a thrombospondin domain [41]. Other approaches to selectively targeting proteinases include biologics such as antibodies to reduce levels of proteinases that promote the disease process [42, 43]. It may also be possible to block cleavage of a key substrate by a proteinases by delivering molecules than bind to and protect the cleavage site of the key substrate of the proteinase and/or modifying sites of the proteinase other than its active site (exosites) involved in substrate binding [44, 45].

##### Knowledge gaps, challenges, and future directions

Future studies should identify all of the proteinase culprits in asthma and their crucial activities in regulating airway pathologies. In particular, we know little about the activities of proteinases in regulating the chronic airway remodeling events that contribute to morbidity in asthma patients [46]. To facilitate the selection of proteinases to be targeted therapeutically, it will be crucial to determine whether proteinases linked to asthma have beneficial or harmful activities in other diseases. In this respect, it is noteworthy that some MMPs that promote AAI and AHR in mice (MMP-7, and −12) have beneficial activities in promoting host defense against pathogens by activating bactericidal proteins or killing bacteria [47, 48].

There are also gaps in our knowledge about the mechanisms that regulate proteinase expression in asthma. Epigenetic regulation of gene expression has been described in asthma, [49] but little is known about epigenetic regulation of proteinases in asthma. Epigenetic regulation mechanisms include gene promoter (de)methylation, histone (de)acetylation and (de)methylation, and the expression of small non-coding RNAs such as micro-RNAs that increase degradation of mRNA transcripts [49–51]. One study linked changes in micro-RNA expression in lungs from allergen-treated mice to alterations in MMP expression [52]. If epigenetic regulation of proteinases is confirmed in asthmatic airways, approaches to epigenetically up regulate the expression of proteinases that reduce the disease expression or to silence the expression of proteinases that promote pathologies in asthmatic airways, represents a promising avenue for future therapeutic intervention in asthma [53–55].

### Blood biomarker changes in COPD patients (Caroline A. Owen)

#### Key points

1. A biomarker can be defined as a characteristic that is objectively measured and evaluated as an indicator of normal biologic processes, pathogenic processes, or pharmacologic responses to a therapeutic intervention.
2. Biomarkers could assist with the management of COPD patients by identifying smokers at risk for developing the disease, assisting with the diagnosis of patients with early-stage disease, phenotyping of COPD patients, selecting subgroups of COPD patients that are most likely to respond to new therapies for clinical trials, and monitoring responses to therapy.
3. Biomarkers studies for COPD can be classified into several groups: 1) cellular; 2) proteomic; 3) genomic; 4) transcriptomic; and 5) metabolomic biomarkers.
4. Currently we lack predictive or prognostic biomarkers that can be measured in biologic samples and have been validated for managing COPD patients or monitoring their responses to therapy.
5. Based upon the current literature, combinations of biomarkers coupled with clinical parameters likely will be the most useful for predicting the course of the disease and its response to therapy in individual patients.

#### Background

COPD is currently the 4^th^ most common cause of mortality [56]. Current therapies improve symptoms but do not alter the course of COPD. Most new therapies that have been tested thus far for COPD have not been shown to have efficacy in clinical trials [57–59]. There are many barriers to the development of more effective therapies for COPD including inadequate phenotyping of COPD patients due to heterogeneity of the disease (emphysema versus airway disease), and failure to select subgroups of patients with phenotypes that are most likely to respond to the therapy. Also, we lack methods to measure COPD activity versus severity. Currently, a single clinical biomarker, the forced expiratory volume in one second [FEV_1_] is used to diagnose, classify, and measure responses to therapy. However, FEV_1_ measurements are often variable and slow to change in response to therapy. Other clinical parameter used measure the effectiveness of therapies (e.g., mortality or exacerbation frequency) may not be very sensitive and/or require the recruitment of large numbers of subjects to show a treatment effect. New biomarkers for COPD have recently been evaluated using high throughput “omics” approaches that may address some of these barriers.

#### Biomarkers and their potential to improve the management of COPD patients

The NIH defines a biomarker as a characteristic that is objectively measured and evaluated as “an indicator of normal biologic processes, pathogenic processes, or pharmacologic responses to a therapeutic intervention” [60]. A clinically-useful biomarker must be reproducible in stable disease, and longitudinal assessments must confirm that a single sample gives a representative result in clinical practice. Biomarkers could improve the management of COPD patients by: 1) aiding the diagnosis of COPD; 2) phenotyping COPD patients for research studies to better understand the disease processes; 3) predicting rate of progression of the disease; 4) identifying sub-groups of COPD patients that might respond to new therapies; and 5) more rapidly and accurately monitoring responses to existing and new therapeutic strategies especially in clinical trials. Biomarkers that have been studied for COPD can be divided into several different groups: 1) cellular; 2) proteomic; 3) genomics; 4) transcriptomic; and 5) metabolomic biomarkers.

#### Cellular biomarkers

##### Sputum leukocytes

Sputum polymorphonuclear (PMN) leukocyte counts are increased in COPD patients and smokers [61, 62]. Sputum PMN counts have been quantified reliably in multicenter COPD clinical trials using standardized methods, and are a relatively stable biomarker in COPD patients [61, 63]. The percentage of PMNs in sputum samples correlates weakly with COPD severity and health status scores, [61] but not with emphysema severity, systemic inflammatory markers, acute exacerbation (AECOPD) frequency, or lung function decline [56]. In another study of smokers, the percentage of sputum PMNs was higher in those with airflow obstruction, chronic mucus hyper-secretion, and rapid rate of decline in lung function [57].

##### Blood leukocytes

COPD patients with high (>2 %) blood eosinophil counts are at increased risk for severe AECOPD and increased mortality from AECOPD [64]. Blood eosinophil counts are simple to assess and are repeatable [65, 66]. COPD patients with blood eosinophil counts > 2 % have reduced AECOPD rates when treated with inhaled steroids, [67] and their AECOPD respond to oral prednisolone therapy [65, 68]. Blood eosinophil counts may serve as a biomarker of asthma-COPD overlap syndrome (ACOS) which is characterized by higher reversibility of airflow obstruction, greater responses to inhaled corticosteroids than usual COPD patients, and eosinophilic airway and systemic inflammation [69].

#### Proteomic biomarkers

Many proteins detected in plasma samples distinguish individuals with COPD from controls and/or are linked to clinical outcomes or phenotypes. These include markers of systemic inflammation (fibrinogen, CRP, IL-6, and IL-8) and lung-derived or pneumoproteins [surfactant protein C (SP-D) chemokine (CC) ligand 18 (CCL-18) and Club cell protein-16 (CC-16)] and other biomarkers such as soluble receptor for advanced end glycation products (sRAGE).

##### Systemic protein biomarkers

Fibrinogen, CRP and IL-6 are acute phase proteins that are synthesized by hepatocytes. Plasma fibrinogen levels are the most robust biomarker for COPD identified so far in terms of relative longitudinal stability, and levels are significantly associated with symptoms, exercise capacity, AECOPD rates, the BODE index, and mortality [70] [71]. Elevated CRP plasma levels are associated with reduced lung function, [72] lower exercise capacity, [73, 74] higher risk of future AECOPD [75] and higher risk of COPD-related hospitalizations [76]. Elevated CRP levels are positively associated with all-cause [77, 78] and COPD-related mortality [79]. Elevated plasma levels of IL-6 (and also fibrinogen and CRP) were independently associated with mortality over 3 years in the Evaluation of COPD Longitudinally to Identify Predictive Surrogate Endpoints (ECLIPSE) COPD cohort after adjusting for clinical variables that predict death in COPD patients [70].

##### Pneumo-proteins

SP-D is produced by type II pneumocytes and Club cells. In the ECLIPSE cohort, SP-D levels were higher in COPD patients than healthy smokers and higher plasma levels predicted AECOPD but not mortality [80]. CC16 is produced by Club cells and has anti-inflammatory activities thereby protecting mice from cigarette smoke (CS)-induced COPD [81, 82]. Plasma CC16 levels are lower in current than former smokers with Global Initiative for Chronic Obstructive Lung Disease (GOLD) stage II-III [83]. Plasma CC16 levels in former smokers with COPD were indirectly (and weakly) related to COPD severity [83]. CC16 plasma levels were indirectly related to rate of decline in lung function over 9 years in patients with mild to moderately-severe COPD in the Lung Health Study (LHS) [84]. CCL18 (or PARC) is predominantly produced in the lungs by macrophages and dendritic cells. Elevated plasma CCL18 levels have been associated with reduced FEV_1_ in COPD patients, higher BODE scores [85], more frequent AECOPD [86], and are independently associated with lung function and mortality [87].

##### sRAGEs

RAGE signaling is linked to endothelial dysfunction in diabetes and metabolic syndrome. A soluble form of RAGE (sRAGE) is generated by proteolytic shedding of RAGE from cell surfaces [88]. sRAGE serves as a decoy receptor that binds to RAGE ligands preventing them from signaling via cell surface RAGE. Plasma sRAGE levels are reduced in COPD patients and levels are inversely related with high resolution computerized tomography (HRCT) scan-defined emphysema [89].

#### Genomic markers

Genetic markers include single nucleotide polymorphisms (SNPs) which have been linked to COPD by genome-wide association studies (GWAS). GWAS support a genetic basis for smoking behavior, susceptibility to develop COPD, and different COPD phenotypes. SNPs in chromosomes 2q21 and 6p21 have been linked to age at smoking initiation, and SNPs in cytochrome P450 genes to lifetime mean number of cigarettes per day, current number of cigarettes smoked per day, and smoking cessation [90]. Several genomic regions are associated with COPD susceptibility at genome-wide significance including family with sequence similarity-13, member A (FAM13A; a gene of unknown function), hedgehog interacting protein (HHIP), a negative regulator of the hedgehog signaling pathway, the cholinergic nicotinic acetylcholine receptor, CHRNA3/5, iron regulatory protein-2 (IREB2), Ras And Rab Interactor-3 (RIN3, which is involved in endocytosis), transforming growth factor-β2, matrix metalloproteinase-12 (MMP-12), AGER, and a region on chromosome 19 [91–93]. Some of these genetic variants are linked to COPD phenotypes including: 1) airflow limitation [CHRNA3/5, IREB2, HHIP (39)]; 2) emphysema (CHRNA3/5, IREB2, TGFB2, MMP-12; [91, 93] 3) fat-free body mass; 4) exacerbation frequency; and 5) systemic disease (HHIP) [94]. Kim et al. linked SNPs in the CC16 and SP-D loci to altered circulating biomarker levels of CC16 and SP-D [95].

The function of some of the protein products of loci that have been linked by GWAS to COPD susceptibility have been linked to COPD pathogenesis by studying mice deficient in these proteins in the CS exposure models of COPD. For example, MMP-12 promotes emphysema development in mice by degrading lung elastin and generating fragments that are chemotactic for monocytes [96, 97]. HHIP protects mice from CS-induced emphysema by reducing lymphocyte numbers and activation on the lung, and reducing the number and size of lymphoid follicles. [98] RAGE promotes emphysema development possibly by increasing the lung inflammatory response to CS [99].

#### Metabolomic biomarkers

There is increased turnover of proteins and lipids in COPD patients that can be detected in serum and urine samples [100]. COPD patients have reduced serum levels of lipoproteins and N,N-dimethylglycine, and branched-chain amino acids (BCAAs) and increased serum levels of glutamine, phenylalanine, 3-methylhistidine and ketone bodies when compared with control subjects [100, 101]. BCAAs, their degradation products, 3-methylhistidine, ketone bodies, and triglycerides correlated negatively with cachexia and positively with systemic inflammation [100]. Another study reported that COPD patients had reductions in sphingomyelins that were associated with emphysema phenotype, and linked elevations in glycosphingolipids to severe COPD exacerbations [102]. COPD patients also have decreased urinary 1-methylnicotinamide, creatinine and lactate, but increased urinary levels of acetate, ketone bodies, carnosine, m-hydroxyphenylacetate, phenylacetyglycine, pyruvate and a-ketoglutarate [101].

#### Transcriptomic biomarkers

Gene expression studies in sputum and blood samples have linked a number of gene expression profiles to the presence of COPD and/or to COPD phenotypes.

##### Sputum studies

Gene array profiling in sputum samples from GOLD stage 2–4 COPD ex-smokers in the ECLIPSE cohort reported changes in 277 genes associated with GOLD stage II vs. III-IV, and 198 genes with changes linked to emphysema severity [103]. Eleven of these genes were replicated in a second COPD cohort using real-time PCR methods [103]. Four distinct and clinically-meaningful combinations of clinical characteristics (especially airflow obstruction but also emphysema severity, plasma fibrinogen levels, chronic production of sputum, body mass index and age) have been associated with large gene expression differences in sputum samples in the ECLIPSE cohort [104]. Gene expression profiling on induced sputum samples from the ECLIPSE COPD study identified functional effects (expression quantitative trait loci of known susceptibility genes including IREB2 and CHRNA5) [105].

##### Blood studies

Using microarrays on blood samples in the ECLIPSE cohort, 150 genes were differentially expressed in frequent exacerbators versus non-exacerbators including down regulation of lymphocyte signaling genes and up regulation of genes involved in apoptosis [106].

#### Biomarker combinations

Combinations of biomarkers have stronger predictive power than analysis of single proteins. The fibronectin/CRP ratio was inversely related to all-cause and cardiovascular disease-related mortality in COPD [107]. The systemic inflammome is a panel of 6 systemic inflammatory markers (blood leukocyte counts, CRP, IL-6, IL-8, fibrinogen and TNF-α) linked to COPD outcomes. In a cohort of 1,755 COPD patients, 16 % of COPD patients had persistent systemic inflammation (defined as the presence of two or more abnormal inflammatory markers), and during follow-up had higher all-cause mortality and AECOPD rates than the 30 % without inflammation [77]. Another study reported that increased levels of five inflammation-sensitive plasma proteins (fibrinogen, ceruloplasmin, α_1_-antitrypsin, haptoglobin, orosomucoid) predicted increased hospital admissions rates for COPD patients during 25 years of follow up [108]. Pinto-Plata et al. combined a clinical model (severity of airflow limitation, carbon monoxide transfer factor, functional capacity, the BODE index and AECOPD frequency) with a panel of plasma protein biomarkers (blood leukocyte counts, and plasma IL-6, fibrinogen, CCL18, CRP, IL-8 and SP-D) to predict mortality. Adding IL-6 (but not the other protein markers) to the clinical model independently added predictive power [85].

#### Summary, conclusions, challenges and future directions

Biomarkers may offer relatively non-invasive means for phenotyping COPD patients, assessing disease activity versus severity, and monitoring responses to therapy. Although progress is being made in identification of COPD biomarkers, no single biomarker studied to date is sufficiently powerful to serve as a diagnostic tool for COPD. Plasma fibrinogen levels could be useful to identify those more likely to exacerbate. Combinations of protein biomarkers coupled with clinical parameters may prove to be most useful in COPD. Advancing biomarkers from discovery to clinical practice is challenging. It requires that the biomarker be validated using hundreds of specimens from carefully-phenotyped COPD patients, and reproducible, specific, sensitive, and stable during sample storage. Standard operating procedures need to be developed for sample collection, processing, and storage. Assays for measuring biomarkers need to be validated at different clinical sites. Analysis of clinical samples from carefully-phenotyped COPD patients using high throughput “omics” technology and integration of these results with clinical data may be the optimal approach to develop personalized signatures that can serve as a biomarker of pathobiologic processes unique to each patient, and to assist in managing the patient with effective targeted therapies.

### The problem of hospital re-admission following discharge after the COPD exacerbation

#### Rationale

Patients discharged following an exacerbation of COPD are at high risk for re-hospitalization within 30 days. This has a substantial negative impact, in terms of quality of life and financial costs. For example, in the US, hospitals will not receive reimbursement for COPD patients re-hospitalized within 30 days. This symposium will define the problem and explore ways to reduce subsequent hospitalizations, morbidity and mortality.

### Characteristics of the Hospitalized COPD Patient (Richard ZuWallack)

#### Key points

- Hospitalized patients with COPD frequently had been hospitalized for exacerbations in the preceding year
- A history of previous hospitalizations for exacerbations for COPD is the strongest predictor of subsequent hospitalizations
- Severity of airflow obstruction (FEV_1_ percent-predicted) does not appear to be significantly lower in hospitalized patients compared with series of ambulatory outpatients with COPD
- Certain comorbid conditions, such as heart failure and diabetes, appear to be more prevalent in hospitalized than ambulatory COPD patients
- Following a hospitalization for COPD, patients are at high risk for dying after discharge from the hospital

Chronic obstructive pulmonary disease (COPD) is a progressive condition punctuated by exacerbations. Often these exacerbations are severe and resulted in hospitalization. This discussion will center on the clinical characteristics of the hospitalized COPD patient: Is he/she different qualitatively or quantitatively from that patient who is able to stay out of the hospital? This brief review will approach this question by 2 methods:

1. Through evaluating the clinical characteristics of hospitalized COPD patients and then comparing these characteristics with those of patients remain as outpatients. This method has the advantage of coming closer to what we want to know: Exactly what features, if any, separate a hospitalized patient from the non-hospitalized patient. Unfortunately, this approach is limited by relatively small numbers of subjects the difficulty in getting a good comparator group and seasonal, regional, and healthcare system differences in hospitalization practices.
2. Through evaluating predictors of hospitalization in large series of COPD outpatients. This has the advantage of providing large numbers of subjects evaluated as outpatients; however an in-depth analysis of patient factors is often not done as part of these large series.

Tables 1, 2 and 3 provide a comparison of patient characteristics from 2 series: 1) A relatively large, longitudinal, observational, multicenter study of 606 patients who had been hospitalized for a COPD (Almagro and colleagues); [109] and 2) A very small observational study of hospitalized COPD patients (Chawla and colleagues) [110]. A large, pharmaceutically-sponsored study on stable outpatients with COPD [111] is used as a comparator group.

**Table 1:** Clinical Characteristics of Patients Hospitalized for COPD (ESMI and SFH) Compared with Stable Outpatients (ECLIPSE)

	ESMI (Spain)	SFH (USA)	ECLIPSE
	Hospitalized	Hospitalized	Out-Patients
Number of subjects	606	54	2138
Male (%)	90	36	65
Age (years)	73	70	63
Current smokers (%)	-	34	36
Body mass index (BMI, kg/m2)	28	32	27
Alone at home (%)	13	69	-
1+ hospitalization prev yr for COPD (%)	71	62	22
1+ exacerbation previous year (%)	-	83	47
First hospitalization for COPD (%)	21	-	-
No prior diagnosis of COPD (%)	8	10	-
Alone at home (%)	13	34	-

ESMI, The EPOC en Servicios de Medicina Interna study [109]; SFH, St. Francis Hospital, Hartford, CT USA [110]. Prev, previous; The dashed line indicates no data available for that variable

**Table 2:** Disease severity factors in hospitalized and non-hospitalized patients with COPD

	ESMI (Spain)	SFH (USA)	ECLIPSE
	Hospitalized	Hospitalized	Out-Patients
On O_2_ rx (%)	39	53	-
FEV_1_ % predicted	43	47	48
G2; G3; G4 (%)	45; 44; 11	-	44; 42; 14
FEV_1_/FVC ≥ 0.70 (% of sample)	-	21	
Could not/would not do 6 MWT (%)	-	20	-
6MWT distance (meters)	-	157	All: 370
			G2: 406
			G3: 357
			G4: 290

O_2_, Oxygen; FEV_1_, Forced expiratory volume in one second; FVC, Forced vital capacity

G2, Global Initiative for Obstructive Lung Disease (GOLD) spirometric stage 2 (FEV_1_ ≥ 50 % and < 80 %); G3, GOLD spirometric stage 3 (FEV_1_ ≥ 30 % and < 50 %); G4, GOLD spirometric stage 4 (FEV_1_ < 30 %). 6MWT, six minute walk test

**Table 3:** Comorbid conditions in hospitalized and ambulatory COPD patients

	ESMI (Spain)	SFH (USA)	ECLIPSE
	Hospitalized	Hospitalized	Out-Patients
Ischemic cardiac disease (%)	21	37	15-17
Heart failure (%)	33	33^a^, 16^b^	4-5^a^
Atrial fibrillation (%)	21	-	-
Chronic kidney disease (%)	16	28	-
Peripheral vascular disease (%)	17	-	-
Diabetes (%)	28	24	12^c^
Hypertension (%)	63	40	-
Depression; anxiety (% %)	15; 18		17; 17^c^
Dementia (%)	4	15	-

^a^Diagnosis suggested by brain naturetic protein > 500 units

^b^Clinical diagnosis by cardiologist

^c^Estimated from Reference [180]

Probably the most outstanding statistic from Table 1 (and of this review) is the marked discrepancy in the number of hospitalizations in the two series of hospitalized patients versus that of the outpatient series: 71 % and 62 % (respectively) of the hospitalized patients had been hospitalized at least once for 1 or more COPD exacerbations in the preceding year, while only 22 % of stable outpatients with COPD had been hospitalized for this reason and the preceding year. Thus, the history of previous hospitalizations for COPD exacerbations is a very powerful predictor of subsequent hospitalizations. In this case, history repeats itself. Of course, this association does not give causal factors: Does this reflect greater disease severity in the “frequent flyers,” or does it reflect other potentially important factors, such as adherence to therapy, comorbid conditions, systemic effects of the disease, proper outpatient management of the disease, or access to medical care?

Of note, mean FEV_1_, expressed as a percent of its reference value, was not different in hospitalized and non-hospitalized patients. Furthermore, the percentages of patients in GOLD (Global Initiative for Obstructive Lung Diseases) spirometric stages 2–4 were strikingly similar in the hospitalized patients in Spain and the ambulatory patients evaluated as part of the pharmaceutical study. This suggests that the degree of airflow limitation is less important a factor in hospitalization versus non-hospitalization. Certainly other important factors are causal when it comes to need for hospitalization in COPD. While patients with GOLD stage 2 were most common, this probably reflects the greater numbers of COPD patients with less severe airflow limitation. One potentially-important marker of disease (not just COPD) severity is the six minute walk distance, which independently predicts survival in this disease [112]. The mean walk distance in the small series by Chawla was 157 m, which is considerably lower than the 350 m threshold that predicts long-term survival in COPD patients beginning pulmonary rehabilitation [112] and is considerably lower than that of outpatients (including Gold stage 4 outpatients) in the pharmaceutical company. This decrease in six minute walk distance in hospitalized COPD patients probably reflects the systemic manifestations of the exacerbation and/or co-morbid events that contributed to the hospitalization.

Of interest is the finding in the Chawla series that 21 % of patients admitted with a clinical diagnosis of COPD did not meet spirometric criteria (i.e., an FEV_1_/FVC < 0.70) for COPD on pre-discharge spirometry. While this might be due, in part, to technical issues – incomplete exhalation in some patients, leading to a falsely-high FEV_1_/FVC, it probably also reflects a potential bias of diagnosing a cigarette smoker with acute respiratory symptoms as having COPD when other potential etiologies should be explored.

Table 3 compares comorbid conditions listed in the three series. Perhaps the discrepancies that stand out most are the higher percentage of inpatients with diagnosed heart failure and diabetes. While these differences might reflect shared comorbidities in the COPD population in general [113], this does not explain the higher percentages in the hospitalized patient. Possible reasons behind this observation include increased disease burden leading to the hospitalization, greater scrutiny in the hospital in identifying other medical problems, or misdiagnosis, such as diagnosing heart failure for a COPD exacerbation in a cigarette smoker.

Table 4 evaluates clinical features of the hospitalized COPD patient from a different perspective. Selected data from the ECLIPSE study, which followed a large number of stable outpatients with COPD longitudinally over three years, are presented here [111, 114].

**Table 4:** Patient Characteristics in Outpatients with COPD that may Predict Re-Hospitalization

	Not hospitalized	Hospitalized
	n = 1,468	n = 670
Age (years)	63	64
Percent female	35	35
BMI (kg/m2)	27	26
Current smoker	37	34
mMRC dyspnea ≥ 2 (%)	46	69
Reflux or heartburn (%)	24	29
Hospitalized for exacerbation over prev yr (%)	9	30
History C-V disease (%)	32	37
FEV_1_ % predicted	51	42
6 MWD (m)	383	341
SGRQ total score	45	55

BMI, Body mass index; mMRC, Modified Medical Research Council; C-V: cardiovascular; SGRQ, St. George’s Respiratory Questionnaire

Again, a history of hospitalization for a COPD exacerbation was the strongest predictor of subsequent hospitalization for the same reason. Greater dyspnea, measured by the Modified Medical Research Council instrument [115], and worse health status, measured using the St. George’s Respiratory Questionnaire [116], also appear to be predictive factors. Interestingly, the mean six minute walk distance in these ambulatory patients, which was only a little lower in those eventually hospitalized than in those who remained outpatients, was considerably higher than in the small sample of hospitalized patients. This suggests that a bi-directional causality with regard to functional status and health care utilization: lower functional exercise capacity predicts subsequent hospitalization in COPD and hospitalization has a profound effect on functional exercise capacity.

With respect to mortality risk, the hospitalized COPD patient is a considerably higher risk of dying after hospital discharge than the patient who remains in an outpatient status. For example, in a prospective study of 135 hospitalized patients for COPD, mortality at six months, one year, and two years was 13.4 %, 22 %, and 35.6 %, respectively [117]. Another post-hospitalization COPD study involving 8325 patients admitted beginning in 1991 revealed a 23 % mortality at one year and a 51 % mortality within 5 years. Contrast these data with those from a pharmaceutical study of 5993 COPD outpatients (mean age 65 years; 30 % current-smokers; FEV_1_ 39% predicted) where the mortality over the 4-year treatment period was 12.8 % and 13.6 % in the treatment group and placebo groups, respectively [118].

In summary, the hospitalized patient with COPD has frequently been hospitalized before for this condition, and this factor appears to be the most potent predictor of subsequent hospitalizations in this population. Airflow limitation appears to be a less important factor than the history of previous hospitalizations. Mortality following a hospitalization for COPD is high.

### Reducing Hospital Readmissions Following COPD Exacerbation (Carolyn Rochester)

Hospitalizations for acute exacerbations of COPD are a major cause of morbidity and mortality and incur significant healthcare costs. Readmissions within 30 days of hospital discharge following acute exacerbations of COPD are common, occurring in 17-30 % of cases [119, 120]. In the United States, an estimated one fifth of all Medicare beneficiaries are readmitted within 30 days, with an estimated annual cost of more than 15 billion dollars [119]. Since 2009, the 30-day readmission rates for congestive heart failure, pneumonia, and myocardial infarction have been reported as a quality performance measure within the US Affordable Care Act; [121] COPD readmission rates have since been added to this list [122]. As of fiscal year 2015, the Centers for Medicare and Medicaid Services has begun penalizing hospitals by decreasing financial reimbursement to those with high rates of unplanned readmission following hospitalizations for COPD exacerbation [123]. As such, identifying the factors associated with increased risk of readmission and patients with these risks is important so as to try to identify treatment plans that will reduce individuals’ risk, improve patient care and in turn reduce healthcare costs.

Numerous factors are associated with an increased risk of hospital readmission for patients with COPD [123–128]. Prior exacerbation history and medical comorbidities are among the most important risks. Other patient-related factors include more severe airflow obstruction, use of supplemental oxygen, older age, low socioeconomic status, marital status, low physical activity levels, functional disability, and impaired quality of life [128, 129]. Healthcare provider-related factors include lack of prescription of short- or long-acting bronchodilator or inhaled corticosteroid within 30 days of discharge, or history of prescription of oral corticosteroid or antibiotic on discharge [123]. Health-system-related factors include hospital length of stay less than 2 or more than 5 days, lack of timely patient follow-up after discharge, and suboptimal transitions of care [123]. However, it is difficult to predict readmission risk for individual patients. A variety of readmission risk prediction models have been published; most, however perform poorly, possibly in part due to insufficient inclusion of factors associated with overall health, functional and socioeconomic status [130]. Thus, it is not possible to reliably predict which patients will require readmission based on any single prediction model.

Therefore, at present, the issue of reducing 30-day hospital readmissions for patients with COPD can be seen as a “good news/bad news” situation. The “good news” is that several individual interventions have been shown to reduce COPD exacerbation risk, which in turn has the potential to reduce the risk of hospitalization. Healthcare professionals can pay rigorous attention to individual patients’ risk factors for readmission (as detailed above) and target interventions to address all of those factors. First and foremost, healthcare professionals must inquire about each patient’s exacerbation history, including the typical symptoms, frequency, prior emergency care visits or hospitalizations; triggers for exacerbations must be identified and addressed (eg. avoidance of allergens or selected environmental conditions and/or treatment of GERD), and “dyspnea crises” related to anxiety and dynamic hyperinflation must be recognized and managed. Patients’ use of maintenance medications for COPD should be assessed and pharmacotherapy adjusted where needed, since inhaled long-acting beta-agonists, long-acting inhaled anticholinergic medications and inhaled corticosteroids reduce the risk of exacerbations by approximately 15-25 % [131–136]. For those with frequent recurrent exacerbations despite optimized inhaled medications, consideration should be given to treatment with other agents shown to reduce exacerbations including macrolide antibiotics [137] or phosphodiesterase-4 inhibitors [138]. Smoking cessation [139] and influenza vaccination [140, 141] also reduce COPD exacerbation risk. Anxiety and depression are common among patients with COPD and are associated with increased hospitalization risk yet are often under-treated; [142, 143] identifying and treating these conditions also has potential to reduce the risk of readmission. One intriguing recent study using the Medicare Premier Research database demonstrated that the provision of oral nutritional supplements during hospitalizations for COPD exacerbation decreased the hospital length of stay and the probability of 30-day readmission among a large sample of Medicare patients over age 65 with a primary hospitalization diagnosis of COPD [144]. Further research is needed to determine the potential role of nutritional supplements in reducing hospitalization risk.

Since persons with respiratory muscle dysfunction (e.g. weakness or mechanical disadvantage related to hyperinflation) may be at greater risk of ventilatory failure in the face of COPD exacerbation, and since respiratory muscle overload at the time of hospital discharge is a factor associated with risk of readmission, [145] interest has arisen in whether use of non-invasive positive pressure ventilation (NIV) during or following COPD exacerbation might reduce subsequent hospitalization risk. Indeed, a retrospective analysis of the outcomes of a multidimensional respiratory therapist-lead program including use of NIV among 397 patients who had been hospitalized more than twice in the previous year demonstrated that the proportion of patients readmitted two or more times decreased to 2.2 % in the year following the intervention (*p* < 0.0001) [146]. However, another study failed to demonstrate any difference in 30-day all-cause or COPD-related readmissions in a retrospective analysis of outcomes of 25, 628 patients hospitalized in the US with acute COPD exacerbation who were treated with NIV for respiratory failure as compared to conventional delivery of mechanical ventilation via endotracheal tube [147]. There may be a subset of patients for whom use of NIV during or after exacerbation reduces readmission risk, but at present the role of NIV in this regard remains uncertain.

Other aspects of post-hospitalization patient management are also important. Patient attendance of follow-up visits with a healthcare professional (primary care provider or pulmonologist) within 30 days of hospital discharge reduces the risk of subsequent emergency department visits [148, 149] and readmission [150]. Pulmonary rehabilitation (PR) improves exercise capacity, reduces symptoms enhances self-efficacy and quality of life and can improve daily physical activity levels [151]. A systematic review of supervised PR implemented early following exacerbations that included 9 randomized controlled trials (patient *n* = 432) demonstrated a 42 % reduction in the chance of hospital admission over median 25 weeks’ follow-up [152]. Other more recent trials have also demonstrated a reduction in exacerbation frequency and hospitalization following participation in PR [153, 154]. One randomized trial involving a six-week rehabilitation intervention begun during hospital admission for an exacerbation of chronic respiratory disease failed to demonstrate reduction in readmission 12 months following the intervention [155]. However, the post-discharge component of PR in this trial was unsupervised, and as such the exercise training stimulus may have been insufficient. Hence, it is not possible to conclude that PR begun during COPD exacerbation and continued post-hospital discharge is ineffective based on this trial. In keeping with the benefits of PR, high physical activity levels [156, 157] and increases in physical activity level over time are associated with reduced risk of hospitalization for patients with COPD.

Self-management programs, which typically include education, action plans, as well as strategies for problem solving, goal setting and resource utilization [158] can help patients acquire skills needed to manage and cope with their disease. Several studies suggest that self-management interventions for patients with COPD can decrease the probability of respiratory-related hospitalization and all-cause hospitalization. However, not all studies of self-management interventions have demonstrated these benefits. Moreover, the optimal timing for them is unclear. While acute exacerbations are often considered an ideal “teachable moment”, a recent systematic review of self-management interventions initiated during an exacerbation of COPD demonstrated no difference at 12 months in quality of life, exercise capacity, primary care use, hospital readmissions or mortality, despite a demonstrated positive effect on patients’ knowledge and management of the exacerbation of COPD [159]. Further work is needed to determine the optimal timing for provision self-management programs for patients with COPD. Multifaceted and integrated care disease management programs that incorporate individually tailored self-management action plans and other aspects of patient support (such as care managers, home visits and call centers) can also reduce readmission risk in selected patient populations and healthcare systems.

The “bad news” is that despite the demonstrated ability of the numerous interventions discussed above to reduce exacerbation and/or hospitalization risk, hospital readmission rates have not decreased substantially in recent years. No single intervention has consistently reduced 30-day hospital readmissions for elderly persons with chronic diseases and no solution has yet been found to significantly reduce the burden of hospital readmission for patients with COPD. There are several possible reasons for this. First, COPD is often misdiagnosed, [160] and many individuals presenting with symptoms suggestive of COPD exacerbation prove to have other conditions such as congestive heart failure or upper airway obstruction. This issue is particularly problematic among underserved patient populations, and misdiagnosis is often associated with the presence of obesity and cardiac disease. Accurate diagnosis is essential for optimized treatment. Misclassification of symptoms and hospitalizations as being related to COPD poses a huge challenge in regard to reducing readmissions following COPD exacerbations, given that many patients do not even have the disease. Second and importantly, COPD is markedly under-diagnosed [161–163], hence individuals at risk for exacerbation and hospitalization are under-recognized and do not have access to risk-reducing therapies. Moreover, for those who do have an accurate diagnosis of COPD, recommended treatments are underutilized. Many practitioners do not adhere to evidence-based treatment guidelines [164, 165], and patient non-adherence to prescribed medical therapies is a major problem [166–169]. Pulmonary rehabilitation is also underutilized; rates of provider referral as well as patient uptake and adherence to PR are low [170–172]. Patients also report multiple barriers to sustaining high levels of daily physical activity [173], and the role of disease-management programs in care of patients with COPD is controversial, since some studies have shown an increase in mortality signal following the intervention [174].

Third, the factors associated with increased risk of hospital readmission are complex and multifaceted, and not all have solutions or are readily amenable to intervention or modification. There likely also are as yet unidentified factors contributing to the readmission risk. Finally, hospital readmissions occur for a myriad of reasons other than COPD exacerbation [175]. As such, interventions such as pharmacotherapies for COPD, pulmonary rehabilitation, self-management programs geared toward reducing COPD exacerbation risk will be ineffective in decreasing these other episodes leading to subsequent hospitalization.

Thus, based on all of these issues, it may ultimately be difficult if not impossible to substantially or consistently reduce patients’ readmission rates following hospitalization for COPD exacerbation. Although the financial penalties posed by insurance payers for high readmission rates may be well intentioned in their effort to improve or streamline care and reduce healthcare costs, payers’ expectations may unrealistic. Nevertheless, healthcare professionals should be encouraged to accurately diagnose COPD among at risk patients, and to utilize evidence-based therapies according to published guidelines. Patients should be encouraged to adhere to these beneficial therapies [176]. Healthcare system inefficiencies must also be addressed. Transitions of care must be improved [177, 178] and provision of integrated care, e.g. in newer models of care delivery such as “patient-centered medical homes” (community-based multidisciplinary care teams and transitional care programs) [179] should be considered. Ongoing efforts to identify means of reducing hospital readmissions, and ongoing dialogue.
